# Editorial: Acute and long-term health issues of occupational exposure to heat and high physical loads

**DOI:** 10.3389/fphys.2023.1304229

**Published:** 2023-10-11

**Authors:** Jennifer Crowe, Beat Knechtle, Daniel Rojas-Valverde

**Affiliations:** ^1^ Central American Institute for Studies on Toxic Substances (IRET), Universidad Nacional de Costa Rica, Heredia, Costa Rica; ^2^ Institute for Family Medicine, Faculty of Medicine, University of Zurich, Zurich, Switzerland; ^3^ Centro de Investigación y Diagnóstico en Salud y Deporte (CIDISAD-NARS), Escuela Ciencias del Movimiento Humano y Calidad de Vida, Universidad Nacional de Costa Rica, Heredia, Costa Rica; ^4^ Sport Injury Clinic, Escuela Ciencias del Movimiento Humano y Calidad de Vida, Universidad Nacional de Costa Rica, Heredia, Costa Rica

**Keywords:** work-related injuries, heat, occupational health, physiological stress, heat stress, ergonomics

In recent years, light has been shed on the dangers of excessive heat and the strenuous demands of physical labor in occupational settings. In the case of heat, some of this attention is driven by increased awareness of climate change, making us more conscious of heat events, which are projected to continue to be more frequent and severe (Intergovernmental Panel on Climate Change, 2023). Unfortunately, some of our awareness also comes from the fact that undesired health outcomes have been documented in working populations (Arbury et al., 2014; Heinzerling et al., 2020; Spector et al., 2023). The same is true for heavy or repetitive movements in the workplace where, despite clear evidence that these exposures can cause severe acute (Lucas et al., 2020) or chronic health outcomes (López-Ruiz et al., 2015), workers continue to be exposed in alarming numbers (Lucas et al., 2020).

For many populations across the globe, the simultaneous combination of high physical demands, inadequate worker protection measures, and high heat exposure continues to be a major challenge for employers, policymakers and, most of all, workers (Morris et al., 2020). Working in heat, particularly with high physical demands can cause a variety of acute health problems ranging from relatively mild heat illness to a dangerous rise in core body temperature that when left untreated, can have catastrophic outcomes (Sorensen and Hess, 2022). These issues are magnified in populations in the informal sector where individuals often juggle more than one job (Venugopal et al., 2021). Even for those in the formal sector, professions including fire fighters, miners, athletes, agricultural laborers, and soldiers, can be in situations where they are quite literally pushing their bodies’ endurance and resistance to the maximum (Lazaro and Momayez, 2021; Carballo-Leyenda et al., 2022; Kim and Lee, 2023). Occupational heat exposure is associated with a number of undesirable health outcomes ranging from headache and nausea to more severe outcomes such as cardiovascular failure (Ebi et al., 2021) or acute kidney injury (Kupferman et al., 2018; Wesseling et al., 2020). Likewise, heavy or repetitive workloads are associated with outcomes ranging from fatigue to acute or chronic musculoskeletal problems (Briggs et al., 2018). Both heavy workload and heat exposure increase the probability that workers will be injured on the job (Fatima et al., 2021; Morrissey et al., 2023).

**FIGURE 1:**
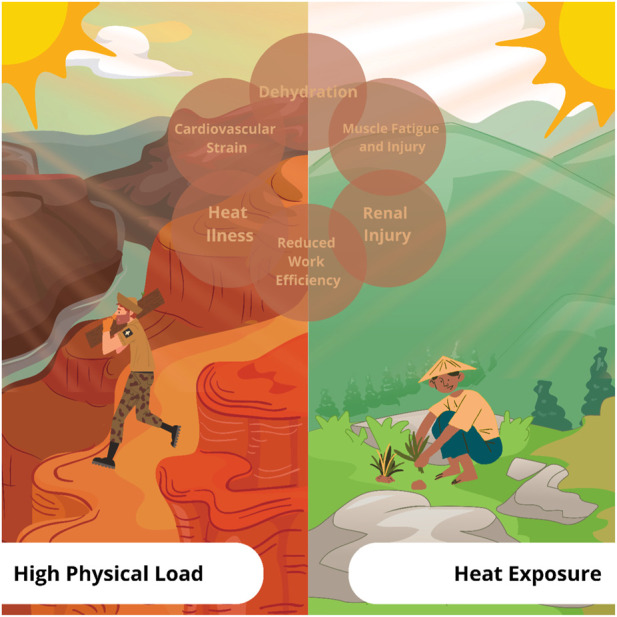
Occupational health issues intersection diagram: Heat exposure and physical strain.

This Research Topic casts a wide net, covering a variety of issues that collectively highlight the nuances of workload and heat exposure while also demonstrating the different areas of science that might lead to better understanding of the physiology of heat exposure or ways to combat health effects resulting from that exposure. Each line of research provides a new bit of knowledge, whether it is the identification of novel biomarkers for the early detection of health issues (Schlader et al., 2019; Burtscher et al., 2022) or the creation of workplace regulations and methods that reduce heat stress (Borg et al., 2021; Morris et al., 2021). The investigation of fatigue recovery approaches and cutting-edge body cooling techniques is the rallying cry for innovation that might reduce occupational risk (Douma et al., 2020; Foster et al., 2020). Additionally, the ability to forecast heat exposure in the face of a changing climate and the continual creation of workforce adaptation techniques are essential for a resilient future (Morris et al., 2020; Habibi et al., 2021; Ioannou et al., 2022). Evidence and solutions are still needed to confront the problems that are triggered when there is an interaction between heat exposure and high physical load in occupational populations (e.g., dehydration, heat stress, renal injury) (see Figure 1).

We invited experts from multiple fields to share results related to health issues resulting from acute and prolonged exposure to heat or heavy physical demands resulting in the four original manuscripts published in this Research Topic. The first manuscript contributed by Arbeille et al., delves into the interplay between isolation and physical activity, demonstrating a lack of physiological effects from a 40-day isolation in a cavern, possibly attributed to sustained physical activity and reduced environmental stress. The second manuscript, contributed by Faricier et al., investigates the acute impact of lower-body cold-water immersion on neuromuscular fatigue after high-intensity exercise, revealing that short-term immersion did not significantly alter neuromuscular function during a maximal intensity fatigue task. The third study from Nguyen et al., illuminates the prevalence of neuropathic pain (e.g., spinal and osteoarthritis pain) among Vietnamese industrial workers, identifying several risk factors related to physical load, emphasizing the importance of ergonomic considerations, and noting other risk factors that were more common in those that reported neuropathic pain including noise, dust and heat. Finally, the fourth manuscript submitted by Wang et al., introduces an innovative approach, employing over-the-counter analgesic creams (e.g., 20% methyl salicylate and 6% L-menthol) as potential tools to enhance skin cooling and core body heat loss during exercise-induced hyperthermia.

Collectively, these manuscripts highlight the multidimensionality of the challenges faced by workers subjected to heat exposure and/or demanding physical loads. The diverse spectrum of outcomes discussed - from arterial wall adaptations to neuromuscular function, from neuropathic pain prevalence to skin cooling interventions - underscores the intricate interplay between the physiological and environmental factors influencing occupational health and therefore the diverse fields of science needed to reduce exposure and treat undesired outcomes when they happen.

As we reflect on the four manuscripts included here together with work published elsewhere in recent months, we encourage researchers from diverse backgrounds to continue to study occupational exposure to heat and high physical loads and to consider populations that may face less obvious risk. For example, heat exposure should be researched in populations including professional athletes and indoor workers in places with internal heat sources such as laundry. Similarly, research on high physical demands is needed for workers in confined spaces or unusual settings such as spacecraft. Finally, research is needed on both topics for workers in the informal sector (López-Ruiz et al., 2015; Julià et al., 2019). We hope this Research Topic encourages academics, professionals, and policymakers to pay special attention to occupational exposures to heat and physical load and to act from their areas of expertise in order to pave the way for safer working conditions.
